# History of Sex Work Is Associated with Increased Risk of Adverse Mental Health and Substance Use Outcomes in Transgender Adults

**DOI:** 10.3390/ijerph192315908

**Published:** 2022-11-29

**Authors:** Ajay Keshav Pandey, Kristie Seelman

**Affiliations:** 1 Department of Biological Sciences, Augusta University, Augusta, GA 30912, USA; 2 School of Social Work, Georgia State University, Atlanta, GA 30302, USA

**Keywords:** transgender, gender diverse, sex work, mental health, substance use

## Abstract

Understanding factors influencing mental health and substance use in transgender and gender diverse people is critical to reducing disparities in this population. We sought to investigate whether a history of sex work was associated with increased prevalence of poor mental health, substance use, and a negative experience within drug and alcohol treatment facilities. We conducted a secondary analysis of the data of 25,204 transgender respondents of the 2015 United States Transgender Survey. We estimated multiple logistic regressions to assess the association between a history of sex work and adverse mental health and substance use outcomes. We then estimated mean prevalence of adverse outcomes by type of sex work. Finally, we performed chi-square analysis to explore differences in mistreatment at drug and alcohol treatment facilities. Respondents with a history of sex work were significantly more likely to have poorer psychological health, suicidality, and substance use after adjusting for covariates. Among those who visited drug and alcohol treatment facilities, those with a history of sex work were significantly more likely to report adverse experiences (26.34% vs. 11.63%). Our findings highlight the increased risk of adverse outcomes in transgender sex workers and emphasize the need for interventions targeting this subgroup of transgender people.

## 1. Introduction

Almost 1 million Americans identify as transgender, an umbrella term for individuals whose gender identity or expression differ from their sex assigned at birth [1]. A growing body of literature has suggested that the stigma, discrimination, and limited opportunities affecting transgender and gender diverse (TGD) people have resulted in wide-reaching health disparities [2]. For example, numerous studies have shown that TGD individuals face an increased burden of mental health problems including anxiety, depression, serious psychological distress, and suicidal thoughts and behavior compared with their cisgender counterparts [3,4,5]. Additionally, TGD people have been shown to have higher rates of problematic use of alcohol, tobacco, and illicit drugs including marijuana [6,7]. Literature has shown that transgender-related discrimination may be a barrier for TGD people attempting to access substance use treatment facilities and may hinder recovery and program completion once in the facilities [8,9]. Furthermore, TGD individuals with intersectional identities, including those who identify as non-White and as disabled, have reported increased discrimination within drug/alcohol treatment facilities and when trying to access other such social services [10,11,12]. Thus, determining potential factors influencing mental health, substance use, and substance use treatment experiences in TGD individuals may help shape policies aimed at reducing mental health and substance use health disparities and improving access to substance use treatment.

Sex workers are individuals who exchange sexual services for money, food, or other goods and services [13]. In the United States, the full-service sex work industry is estimated to generate 14 billion dollars annually, with at least 1–2 million Americans engaging in full-service sex work [13]. Sex workers are often exposed to increased social stigma [14] and violence [15] than their counterparts who do not have a history of sex work. Even more striking, Sprankle and colleagues reported that, following sexual assault, sex-workers were more likely to experience victim blaming (people believing that the sex worker’s actions resulted in assault or that sex workers cannot be assaulted due to the nature of their work) compared to those not engaged in sex work, potentially limiting their access to justice, social services, and healthcare [16]. The increased social stigma and violence experienced by sex workers has been shown to decrease resiliency [17] and increase the prevalence of poor mental health and suicide behaviors in this population [18,19,20]. Sex workers may turn to substance use in an effort to cope with the psychological distress associated with sex work and it associated violence and social stigmatization [20,21]. Interestingly, alcohol and drug use behavior in sex workers was associated with increased prevalence of violence [22,23], which may create a positive feedback loop in which the substance use intended to cope with the psychological distress associated with sex work may in fact increase a sex worker’s exposure to violence and stigma leading to even worse mental health outcomes.

Some TGD individuals view sex work as their only available career path, which may expose them to increased stigma, violence, and discrimination [24,25,26]. A few previous studies have indirectly assessed whether sex work was correlated with increased alcohol and drug abuse in TGD adults by identifying predictors of adverse mental health and substance abuse outcomes [27,28,29,30]. These studies largely utilized small sample sizes and were conducted outside of the United States. Additionally, these studies did not assess the role relevant socioeconomic and demographic covariates played in driving this association nor did they analyze the impact of the type of sex work on outcomes. Furthermore, while it has been suggested that a history of sex work may negatively impact mental health outcomes [31], this relationship is not clear [32,33]. Finally, no studies have investigated how the intersectional identity of being TGD and a sex worker influences treatment within drug and alcohol treatment facilities.

### Research Question

Though a few previous studies have indirectly assessed the association between gender identity, sex work, substance use, and poor mental health, little is known about the role of sex work on mental health and substance use outcomes in TGD adults. We seek to fill in these gaps in knowledge regarding the role of sex work in exacerbating health disparities experienced by TGD individuals by addressing three questions: (1) how is a history of sex work associated with mental health and substance use outcomes in TGD adults after controlling for relevant covariates; (2) how does the prevalence of poor mental health and substance use vary by type of sex work, and (3) how does a history of sex work impact a TGD individual’s experience at a drug/alcohol treatment facility?

## 2. Methods

### 2.1. Data

We utilized the data of the 25,204 transgender and gender diverse individuals of the 27,715 total respondents to the 2015 United States Transgender Survey (USTS) for whom history of sex work, mental health and substance use outcomes, and sociodemographic data was available. Within this sample, the majority were never married (70.37%), were less than 45 years old (82.83%), employed (66.03%), not disabled (72.19%), and identified as white (81.91%). There was a broad range of gender identities, sexual orientations, income and educational levels, and geographic locations represented by our sample of transgender adults. These de-identified data were made available to the second author by the National Center for Transgender Equality for analysis which took place in 2022. As part of the USTS, respondents aged 18 and older were recruited using online convenience sampling or through organizational partners and completed the survey anonymously online in either English or Spanish. More information about the USTS methodology can be found in the report by James et al. [34]. This project was designated as not human subjects research by the IRB of Georgia State University.

### 2.2. Measures

All variables analyzed within this study are summarized within Table 1.

#### 2.2.1. Independent Variable

The history of sex work dichotomous independent variable was constructed through two questions. The first question asked, “Have you ever engaged in sex or sexual activity for money (sex work) or worked in the sex industry (such as erotic dancing, webcam work, or porn films)?” with the answer choices being either yes or no. The second question asked respondents “Have you engaged in sex or sexual activity for any of the following?” with possible selections including food, place to sleep, drugs, and something not listed. Of note, the survey specified that “These are questions about work for pay in the sex industry and sex work” to ensure that respondents knew to distinguish sex work from sex for pleasure/enjoyment (or another reason not related to sex work/trade). If a respondent indicated that they had engaged in sex or sexual activity for money, they were asked to mark all that apply to the question “What type of sex work or work in the sex industry have you ever done?” with potential answer choices: street-based sex work; sex work advertised online; sex work advertised in magazines and newspapers; informal sex work through word of mouth, occasional hookups with dates in my networks, or things like that; escort, call girls, rent boy with an agency; pornography/picture or video; phone sex; webcam work; erotic dancer/stripper; fetish work (domme, sub, switch); or not listed, which we classified as “other” for simplicity.

#### 2.2.2. Mental Health Outcome Variables

Serious psychological distress was measured using the Kessler Psychological Distress Scale (K6), which asks how often in the past 30 days respondents felt: so sad that nothing could cheer them up, nervous, restless or fidgety, hopeless, that everything was an effort, and worthless. Potential responses included: none of the time, a little of the time, some of the time, most of the time, and all of the time (with the answers being coded from 0–4, respectively). The scores were added to produce a scale ranging from 0 to 24. Respondents with a score of 13 or higher (out of 24) were coded as experiencing serious psychological distress.

Respondents were coded as ever having suicidal thoughts if they responded yes to either “At any time in the past 12 months, did you seriously think about trying to kill yourself?” or “At any time in your life, have you seriously thought about trying to kill yourself?”. Respondents were recorded as ever having attempted suicide if they responded yes to either of the following questions: (1) “During the past 12 months, did you try to kill yourself?” or (2) “At any time in your life, did you try to kill yourself?”. If a respondent attempted suicide in their lifetime, they were asked “How many times have you tried to kill yourself in your lifetime?”, which we used to assess whether a respondent had multiple suicide attempts. When asked about suicidal thoughts and behaviors, respondents were provided with the links to the National Suicide Prevention Helpline, the Veterans Crisis Line, and the Trevor Project.

#### 2.2.3. Substance Use Outcome Variables

For the question “How long has it been since you last smoked part or all of a cigarette?”, respondents were marked as current smokers if they responded, “Within past 30 days”. Respondents who indicated that they had smoked part or all of a cigarette in the past 30 days were asked “During the past 30 days, on how many days did you smoke part or all of a cigarette?”. Respondents who indicated 30/30 days were coded as daily smokers, with respondents who indicated they smoked 29 or less days a month being coded as non-daily smokers.

To assess alcohol usage, respondents were asked “During the past 30 days, on how many days did you have 5 or more drinks on the same occasion?”, with drink being defined as a can or bottle of beer, a glass of wine or a wine cooler, a shot of liquor, or a mixed drink with liquor in it. If the respondent replied with “At least one day”, they were coded as a binge drinker. Respondents were coded as currently using other illicit drugs if they responded that they have used any prescription drug not as prescribed or not prescribed to them or any illegal/illicit drug (such as cocaine, crack, heroin, LSD, meth, inhalant like poppers or whippits) within the past 30 days. Respondents were coded as current marijuana users if they responded that they have used marijuana or hashish in the past 30 days. Marijuana use was separated from other illicit drugs as some states, such as Colorado, had legalized marijuana use before the study period, potentially leading to overestimation of illicit drug use due to the contribution of marijuana users.

#### 2.2.4. Experiences at Drug/Alcohol Treatment Facilities

Respondents who indicated that they had visited a drug or alcohol treatment facility within the past year were then asked whether they had been denied equal treatment, were harassed, or were attacked as a result of their trans identity. We coded all individuals who responded that they had one or more of these experiences as having had an adverse experience within a treatment facility, regardless of whether they had indicated they were out as transgender at the drug/alcohol treatment facility.

### 2.3. Statistical Analysis

All statistical analyses were conducted using STATA 17.0 basic edition. Due to the overrepresentation of individuals who were white and 18 years old, we utilized recommended sampling weight using the STATA svy command to correct for this overrepresentation. Multiple weighted univariate logistic regression analyses were conducted to describe the relationship between sociodemographic variables on mental health and substance abuse outcome variables. We then determined the crude and adjusted odds ratios in favor of having adverse mental health and substance abuse outcome variables for those with a history of sex work controlling for sociodemographic variables using weighted univariate and multivariate logistic regression models. Before using sample weights, we reviewed the Pregibbon delta statistic and found no indication of extreme outliers that would significantly influence the model. Next, we describe the prevalence of adverse mental health and substance abuse outcomes on the 2596 respondents who indicated they have engaged in sex work for money by estimating the means and 95% confidence intervals of respondents reporting negative outcomes. Finally, we explored the experience of the 401 respondents who reported going to a drug/alcohol treatment facility. We utilized chi-square analyses to examine associations between having a history of sex work for any reason and having adverse experiences at drug/alcohol treatment facilities.

## 3. Results

Multiple weighted univariate logistic regression analyses were conducted to see whether relevant socioeconomic and demographic variables, including age group, gender identity, and sexual orientation, were associated with study outcome variables, namely serious psychological distress, suicidality, and various forms of substance use. The results show that serious psychological distress, ever having suicidal thoughts, and having a lifetime suicide attempt were associated with having a non-heterosexual sexual orientation, being 18–24 years old, having never been married, having lower education levels, having no income, being unemployed, and identifying as disabled. Substance use (including tobacco, alcohol, and drug use) was associated with being aged 25–44 years old, having never been married, having lower educational levels, and identifying as disabled. Table 2 describes the socioeconomic and demographic characteristics of the study population and the association between background characteristics and the mental health and substance abuse outcome variables.

TGD adults with a history of sex work were more likely to experience serious psychological distress, to report having serious suicidal thoughts, and to have at least one lifetime suicide attempt with adjusted odds ratios of 1.437 (1.180–1.749), 1.669 (1.259–2.212), and 2.091 (1.708–2.560), respectively (see Table 3). Interestingly, among respondents with a lifetime suicide attempt, individuals with a history of sex work were also more likely to report more than one lifetime suicide attempt. TGD individuals with a history of sex work were more likely to self-report using tobacco both within the past month and daily, binge drinking, and using marijuana and other illicit drugs than those without a history of sex work. Table 3 further details the relationship between history of sex work and study outcome variables before and after adjusting for relevant covariates.

Among those with a history of sex work for money, individuals were often involved in more than one type of sex work. The three most prevalent types of sex work respondents engaged in were sex work advertised online (32.05%), informal sex work through word of mouth (37.79%), and webcam work (38.21%). Table 4 presents summary statistics depicting the number of respondents who engaged in each type of sex work, with the mean percentage (and the corresponding 95% CI) of those reporting adverse mental health and substance use outcomes. Those engaged in webcam work were the most likely to report experiencing serious psychological distress and ever having serious suicidal thoughts. Correspondingly, those engaged in phone sex were the most likely to report having at least one attempted suicide and, among those with at least one suicide attempt, to have more than one attempted suicide. There were less clear trends when looking at substance use. Those who reported being an escort with an agency or engaging in other forms of sex work had the highest percentage of respondents with current or daily tobacco use. Those engaged in informal sex work through word of mouth, however, were the most likely to report binge drinking. Respondents engaged in fetish work had the highest marijuana usage and were the most likely to report both binge drinking and non-marijuana illicit substance use. Finally, those engaged in other forms of sex work were the highest users of non-marijuana illicit drugs.

Given the prevalence of drug and alcohol use among TGD individuals, especially TGD sex workers, understanding treatment experiences at drug and alcohol treatment facilities is critical in reducing the prevalence of substance abuse in this population. Table 5 presents summary statistics about adverse experiences at drug and alcohol treatment facilities separated by history of sex work. The percentage of respondents reporting adverse experiences in drug and alcohol treatment facilities was more than double among those with a history of sex work (26.34% vs. 11.16%).

## 4. Discussion

Our results suggest that even after adjusting for relevant covariates, TGD adults with a history of sex work are more likely to experience poor mental health, to exhibit suicidal thoughts and behaviors, and to use alcohol, drugs, and tobacco than their counterparts without a history of sex work. Similarly, Miller and colleagues found that Guatemalan transgender women sex workers were significantly more likely to use illicit drugs and binge drink compared to cisgender males not engaged in sex work [35]. Interestingly, this association held but was not significant when cisgender male sex workers were used as the reference group, suggesting that being a gender minority does not significantly increase the stressors of sex workers. Our findings however implicate having a history of sex work as an added burden to TGD adults who are already under increased stress when compared to their cisgender counterparts. This association is likely mediated through the increased burden of violence, social stigmatization, and sexually transmitted infections (STIs) seen in the sex worker population compared with their peers not engaged in sex work [14,18,35,36].

Given that sex workers engaged in different types of sex work may be exposed to different levels of stigma and violence [37,38], we explore for the first time the prevalence of poor mental health and substance use by type of sex work in TGD adults with a history of sex work for money. We found that individuals engaged in webcam work or phone sex had the highest prevalence of serious psychological distress, suicidal thoughts, and suicidal behaviors. Argento and colleagues suggested that the move from outdoor sex work to online solicitation coincided with increased social isolation due to loss of social support networks among male sex workers [37]. Social support has previously been implicated in mitigating poor mental health and suicidality resulting from discrimination, harassment, and rejection [39], and peer support services have been shown to decrease internalized stigma among sex workers [40]. Thus, losing these social networks may negatively impact the mental health of TGD sex workers. We also found that individuals engaged in different forms of sex work may use different substances to cope with the varying stressors they experience. For example, those engaged in informal sex work had the highest prevalence of binge drinking while those engaged in fetish work had the highest usage of marijuana and the combined binge drinking and non-marijuana illicit drug use. More research is needed to better understand the different stressors experienced by sex workers engaged in different forms of sex work and how these stressors differently impact social networks, mental health, and substance use.

Finally, understanding the experiences of TGD people at substance use treatment facilities may help shape policies aimed at increasing the utilization of these facilities and decreasing the prevalence of substance abuse [41]. Literature has shown that transgender men and women often reported discrimination within drug and alcohol treatment facilities, which often limits their ability to recover [8]. TGD intersectional identities with other minoritized groups including racial minorities and those with disabilities have previously been found to increase the likelihood of experiencing discrimination within treatment facilities [11,12]. We add to this body of literature on the role of intersectionality on discrimination when accessing social services by showing that TGD adults who identify as sex workers are significantly more likely to report adverse experiences, including discrimination and physical violence, within drug and alcohol treatment facilities. This finding is in line with other studies that found sex workers, especially those with a transgender identity, face increased discrimination and stigma in other healthcare settings outside of substance use treatment facilities [26,42,43]. Increasing sensitivity toward the unique needs of transgender sex workers may be key in addressing the increased problem of substance abuse in this population.

### 4.1. Limitations

The large sample of TGD adults and the relatively large number of sex workers captured by this study is a major strength of this work. Furthermore, our study, unlike many previous studies, included individuals who identify as nonbinary and crossdressers rather than only those who identify as transgender. The present study is however subject to several limitations. First, we are unable to establish a causal relationship between having a history of sex work and any of the study outcomes due to the cross-sectional nature of survey data. Second, the use of convenience sampling prevents the generalization of study findings to the American TGD population. Third, the survey data was from 2015, before the COVID-19 pandemic. Recent evidence suggests that the COVID-19 pandemic and its associated lockdowns have led to a shift from in-person to virtual forms of sex work [44,45]. Furthermore, sex workers during the pandemic may face increased financial hardship, food insecurity, and homelessness which may impact their mental health and substance use [44,46]. The United States Transgender Survey is currently accepting survey responses for the 2022 wave of the survey. Results from this study should be updated following the publication of this data to represent potential changes resulting from the COVID-19 pandemic. Additionally, state-level policies regarding marijuana use, in particular recreational marijuana use, has changed since 2015 which may impact prevalence of usage. Fourth, there may be underreporting of adverse experiences within drug and alcohol treatment facilities. The USTS asks respondents about adverse experiences within the context of their transgender identity, which opens the potential that respondents who experienced discrimination related to racial identity, sex work, or other reasons may not respond as having adverse experiences. We attempted to minimize this underreporting by including respondents who indicated adverse experiences but also reported not being out, or having disclosed their trans identity, within the alcohol and drug treatment facility.

### 4.2. Theoretical Implications

In the present study, we find that having a history of sex works increases the prevalence of serious psychological distress, suicidality, substance use, and negative experiences within substance use treatment facilities in TGD adults. We also found that the prevalence of adverse mental health and substance use outcomes varied by the type of sex work the respondent engaged in. Evidence has suggested that a combination of decriminalization and reductions in stigma surrounding sex work are crucial in mitigating the mental health risks of sex work [40,47]. Given that varying forms of sex work may carry different health risks and varied levels of stigma, our findings highlight the need for additional research to help inform policies and guide public health interventions seeking to reduce the disparity in mental health and substance use outcomes in TGD adults, especially TGD sex workers. Additionally, the increased burden of poor mental health and substance use for transgender sex workers compared with their non-sex worker transgender peers may be the result of increased rates of interpersonal violence and sexually transmitted diseases seen in this subgroup. The creation of mental health and substance use services that specifically address violence experienced by sex workers and multi-level violence interventions may have a positive impact on reducing the prevalence of violence, poor mental health, substance use, and STIs [20,48,49].

Recently, there has been a call for an increase in culturally sensitive strategies to improve mental health and substance use treatment programs and facilities to help reduce barriers to healthcare for TGD adults [50,51]. A qualitative study analyzing the counseling experience of TGD adults found that those with intersectional identities, such as identifying as TGD and a racial minority, often felt as though their providers did not understand their unique experiences due to holding multiple identities [52]. Sex worker participants of another qualitative study reported experiencing pervasive stigma relating to their profession, which had implications for disclosure of their sex worker identity and treatment by mental health professionals [40]. The results of our study also support the development of cultural competency training for healthcare providers, especially those engaged in mental health and substance use treatment, regarding working with the intersectional identities of TGD sex workers in order to increase utilization of these services.

## 5. Conclusions

We find that having a history of sex work increased the odds of having serious psychological distress, suicidality, and substance use even after adjusting for covariates. We also found that transgender sex workers were more likely to report adverse experiences within drug and alcohol treatment facilities compared with their counterparts without a history of sex work. The results of this study have major implications for shaping policies aimed at reducing substance use and improving the mental health of TGD adults, especially those with a history of sex work.

## Figures and Tables

**Table 1:** Summary of Study Variables.

Independent Variable	History of Sex Work for Any Reason	
Outcome Variables	Serious Psychological Distress (Kessler 6 score > 13) History of serious suicidal thoughts and an attempted suicide History of more than one suicide attempt (for respondents who reported at least one attempted suicide) Current tobacco use Daily tobacco use (for respondents who reported being current users)	Binge drinking within past 30 days Marijuana and other illicit drug use within past 30 days Binge drinking and non-marijuana illicit drug Adverse experience (denied equal treatment, harassment, physical attack) at drug/alcohol treatment facility (for respondents who indicated usage of these facilities)
Covariates	Gender identity Sexual orientation Age group Marital status Census region	Education level Individual income level Employment status Disability status Race and ethnicity

**Table 2:** Estimates of Odds Ratio of Experiencing Adverse Mental Health and Substance Use Outcomes across Sociodemographic Characteristics.

						Mental Health Outcomes			Substance Use Outcomes		
Background Characteristic	*n* (%)	Serious Psychological Distress	Ever Had Serious Suicidal Thoughts	Ever Attempted Suicide	Ever Had More than One Suicide Attempt	Current Tobacco Use	Daily Tobacco Use	Binge Drinking	Marijuana Use	Other Illicit Drug Use	Binge Drinking + Non-Marijuana Illicit Drug Use
**Gender Identity**											
Man/Trans Man	7449 (29.55)	Ref.	Ref.	Ref.	Ref.	Ref.	Ref.	Ref.	Ref.	Ref.	Ref.
Woman/Trans Woman	8512 (33.77)	0.795 ***	0.682 ***	0.781 ***	0.813	0.815 **	1.098	0.767 ***	0.787 **	0.774 **	0.645 **
		(0.671–0.942)	(0.544–0.856)	(0.663–0.920)	(0.623–1.062)	(0.674–0.984)	(0.861–1.401)	(0.644–0.914)	(0.655–0.946)	(0.601–0.997)	(0.453–0.920)
Non-Binary/Genderqueer	8606 (34.15)	1.787 ***	1.097	0.819 **	1.048	0.646 ***	0.423 ***	0.927	1.032	1.252 *	1.231
		(1.526–2.093)	(0.876–1.373)	(0.699–0.960)	(0.832–1.321)	(0.535–0.779)	(0.322–0.557)	(0.783–1.098)	(0.865–1.231)	(0.962–1.628)	(0.843–1.797)
Cross-Dresser	637 (2.53)	0.331 ***	0.184 ***	0.179 ***	0.314 **	0.696	1.044	1.342	0.352 ***	0.198 ***	0.154 ***
		(0.191–0.575)	(0.123–0.274)	(0.103–0.312)	(0.115–0.856)	(0.407–1.190)	(0.510–2.137)	(0.868–2.077)	(0.225–0.550)	(0.112–0.351)	(0.065–0.365)
**Sexual Orientation**											
Heterosexual/Straight	3041 (12.07)	Ref.	Ref.	Ref.	Ref.	Ref.	Ref.	Ref.	Ref.	Ref.	Ref.
Asexual	2696 (10.70)	2.253 ***	1.651 ***	1.132	0.939	0.495 ***	0.646 *	0.503 ***	0.650 *	0.753	0.301 ***
		(1.653–3.070)	(1.165–2.340)	(0.847–1.513)	(0.563–1.568)	(0.345–0.710)	(0.408–1.022)	(0.357–0.708)	(0.423–1.000)	(0.422–1.341)	(0.162–0.558)
Bisexual	3725 (14.78)	1.308 *	1.386 **	0.964	0.897	0.867	0.972	1.159	1.039	0.775	0.724
		(0.960–1.783)	(1.015–1.895)	(0.733–1.268)	(0.548–1.469)	(0.632–1.189)	(0.653–1.447)	(0.859–1.564)	(0.787–1.371)	(0.523–1.148)	(0.423–1.239)
Gay/Lesbian/Same-Gender Loving	4228 (16.78)	1.416 **	1.758 ***	1.037	1.081	0.721 **	0.831	0.842	1.032	0.949	0.835
		(1.061–1.889)	(1.339–2.307)	(0.806–1.334)	(0.686–1.704)	(0.542–0.960)	(0.581–1.190)	(0.649–1.093)	(0.778–1.371)	(0.642–1.404)	(0.494–1.412)
Pansexual	4585 (18.19)	2.911 ***	3.381 ***	1.791 ***	1.058	0.902	0.785	1.525 ***	1.858 ***	1.647 **	2.040 **
		(2.175–3.896)	(2.567–4.452)	(1.380–2.323)	(0.629–1.779)	(0.687–1.185)	(0.568–1.085)	(1.149–2.023)	(1.398–2.469)	(1.074–2.525)	(1.161–3.584)
Queer	5266 (20.89)	2.289 ***	2.764 ***	1.273 **	1.048	0.996	0.544 ***	1.607 ***	2.587 ***	1.991 ***	2.394 ***
		(1.732–3.026)	(2.129–3.588)	(1.001–1.620)	(0.669–1.641)	(0.768–1.292)	(0.394–0.751)	(1.260–2.050)	(2.028–3.300)	(1.341–2.956)	(1.385–4.141)
Not Listed	1663 (6.60)	2.134 ***	1.794 **	1.180	1.818 **	0.523 ***	0.538 **	0.751 *	1.040	1.261	1.446
		(1.494–3.048)	(1.147–2.807)	(0.829–1.679)	(1.038–3.184)	(0.358–0.765)	(0.318–0.913)	(0.538–1.047)	(0.717–1.509)	(0.753–2.111)	(0.748–2.796)
**Age Group**											
18–24	10,703 (42.47)	Ref.	Ref.	Ref.	Ref.	Ref.	Ref.	Ref.	Ref.	Ref.	Ref.
25–44	10,172 (40.36)	0.455 ***	0.681 ***	1.070	1.034	1.495 ***	2.750 ***	1.316 ***	1.288 ***	1.222 *	1.130
		(0.406–0.509)	(0.580–0.800)	(0.954–1.199)	(0.863–1.238)	(1.311–1.705)	(2.254–3.355)	(1.164–1.488)	(1.129–1.470)	(0.999–1.495)	(0.862–1.481)
45–64	3648 (14.47)	0.177 ***	0.366 ***	0.722 ***	0.692 **	1.217 *	3.226 ***	0.812 **	0.632 ***	0.564 ***	0.430 ***
		(0.144–0.217)	(0.302–0.444)	(0.608–0.857)	(0.503–0.950)	(0.999–1.483)	(2.541–4.096)	(0.666–0.989)	(0.521–0.765)	(0.436–0.730)	(0.296–0.624)
65+	681 (2.70)	0.070 ***	0.184 ***	0.286 ***	0.558 *	0.445 ***	1.378	0.474 ***	0.342 ***	0.148 ***	0.086 ***
		(0.044–0.113)	(0.142–0.238)	(0.208–0.395)	(0.310–1.004)	(0.301–0.656)	(0.863–2.198)	(0.327–0.686)	(0.239–0.489)	(0.089–0.246)	(0.035–0.213)
**Marital Status**											
Married/CU/RDP	4577 (18.16)	Ref.	Ref.	Ref.	Ref.	Ref.	Ref.	Ref.	Ref.	Ref.	Ref.
Widowed/Divorced/Separated	2891 (11.47)	1.033	1.121	1.321 **	1.097	1.817 ***	1.994 ***	1.304 **	1.178	0.701 *	0.716
		(0.811–1.315)	(0.877–1.433)	(1.068–1.634)	(0.748–1.609)	(1.404–2.350)	(1.431–2.779)	(1.007–1.688)	(0.907–1.530)	(0.487–1.008)	(0.419–1.224)
Never Married	17,736 (70.37)	2.819 ***	1.936 ***	1.653 ***	1.221	1.588 ***	1.124	1.367 ***	1.712 ***	1.577 ***	1.848 ***
		(2.347–3.386)	(1.567–2.391)	(1.390–1.965)	(0.901–1.654)	(1.288–1.957)	(0.847–1.492)	(1.140–1.639)	(1.399–2.095)	(1.154–2.156)	(1.177–2.902)
**Census Region**											
Northeast	5192 (20.60)	Ref.	Ref.	Ref.	Ref.	Ref.	Ref.	Ref.	Ref.	Ref.	Ref.
Midwest	5281 (20.95)	1.068	0.998	1.210 *	1.057	1.185	1.672 ***	0.979	0.680 ***	0.947	0.867
		(0.859–1.328)	(0.752–1.324)	(0.966–1.517)	(0.703–1.591)	(0.897–1.564)	(1.213–2.306)	(0.757–1.266)	(0.515–0.897)	(0.662–1.356)	(0.575–1.308)
South	6922 (27.46)	1.120	1.131	1.098	1.313	0.984	1.354 **	0.978	0.753 **	1.033	1.095
		(0.902–1.391)	(0.867–1.474)	(0.879–1.372)	(0.900–1.917)	(0.755–1.284)	(1.006–1.822)	(0.758–1.261)	(0.581–0.977)	(0.735–1.451)	(0.668–1.796)
West	7809 (30.98)	0.970	1.018	1.002	1.167	1.105	1.272	0.924	1.110	1.226	0.874
		(0.795–1.183)	(0.783–1.323)	(0.812–1.238)	(0.772–1.764)	(0.845–1.445)	(0.928–1.743)	(0.719–1.189)	(0.873–1.411)	(0.919–1.635)	(0.595–1.283)
**Education Level**											
High School or Less	3810 (15.12)	Ref.	Ref.	Ref.	Ref.	Ref.	Ref.	Ref.	Ref.	Ref.	Ref.
Some College/Associate’s Degree	11,674 (46.32)	0.901	1.372 ***	0.946	0.790	0.752 ***	0.625 ***	0.946	0.992	1.013	1.089
		(0.762–1.066)	(1.097–1.715)	(0.796–1.124)	(0.573–1.091)	(0.623–0.906)	(0.499–0.783)	(0.779–1.150)	(0.821–1.197)	(0.779–1.319)	(0.746–1.590)
Bachelor’s Degree	6443 (25.56)	0.536 ***	0.974	0.587 ***	0.537 ***	0.515 ***	0.403 ***	1.009	0.858	0.802	0.843
		(0.449–0.639)	(0.777–1.222)	(0.491–0.703)	(0.385–0.749)	(0.421–0.631)	(0.308–0.527)	(0.827–1.231)	(0.704–1.046)	(0.606–1.061)	(0.564–1.258)
Graduate or Professional Degree	3277 (13.00)	0.308 ***	0.706 ***	0.462 ***	0.528 ***	0.339 ***	0.245 ***	0.698 ***	0.650 ***	0.586 ***	0.522 ***
		(0.249–0.382)	(0.560–0.891)	(0.379–0.563)	(0.367–0.760)	(0.271–0.423)	(0.181–0.331)	(0.560–0.870)	(0.524–0.806)	(0.430–0.799)	(0.337–0.810)
**Individual Income Level**											
No Income	3633 (14.41)	Ref.	Ref.	Ref.	Ref.	Ref.	Ref.	Ref.	Ref.	Ref.	Ref.
$1–$9999	7227 (28.67)	0.776 *	0.928	1.268 *	1.274	1.341 *	1.636 **	1.229	0.996	0.887	0.794
		(0.582–1.035)	(0.538–1.599)	(0.963–1.669)	(0.817–1.986)	(0.992–1.811)	(1.103–2.427)	(0.868–1.739)	(0.716–1.387)	(0.545–1.443)	(0.381–1.651)
$10,000–$24,999	5662 (22.46)	0.417 ***	0.834	1.029	1.198	1.749 ***	2.540 ***	1.559 **	1.060	0.834	0.878
		(0.319–0.546)	(0.493–1.411)	(0.796–1.331)	(0.821–1.749)	(1.303–2.347)	(1.754–3.678)	(1.101–2.209)	(0.762–1.474)	(0.507–1.370)	(0.403–1.914)
$25,000–$49,999	4172 (16.55)	0.213 ***	0.608 *	0.613 ***	0.830	1.178	1.489 **	1.376 *	0.730 *	0.575 **	0.710
		(0.162–0.282)	(0.366–1.011)	(0.475–0.792)	(0.584–1.178)	(0.871–1.593)	(1.017–2.180)	(0.983–1.927)	(0.529–1.006)	(0.352–0.939)	(0.333–1.515)
$50,000+	4510 (17.89)	0.137 ***	0.366 ***	0.402 ***	0.564 ***	0.660 ***	1.050	1.228	0.486 ***	0.420 ***	0.500 *
		(0.102–0.186)	(0.222–0.604)	(0.309–0.521)	(0.384–0.828)	(0.499–0.874)	(0.735–1.500)	(0.883–1.710)	(0.353–0.668)	(0.257–0.687)	(0.229–1.092)
**Employment Status**											
Employed	16,642 (66.03)	Ref.	Ref.	Ref.	Ref.	Ref.	Ref.	Ref.	Ref.	Ref.	Ref.
Unemployed	3244 (12.87)	2.664 ***	1.681 **	1.606 ***	1.145	1.084	0.997	1.024	1.140.	1.512 **	1.421
		(2.105–3.370)	(1.096–2.578)	(1.271–2.031)	(0.829–1.583)	(0.833–1.412)	(0.690–1.440)	(0.779–1.347)	(0.879–1.479)	(1.025–2.230)	(0.793–2.545)
Out of Labor Force	5318 (21.10)	1.371 ***	0.973	1.389 ***	1.167	1.069	1.347 **	0.634 ***	0.928	0.898	0.725
		(1.142–1.647)	(0.781–1.212)	(1.156–1.669)	(0.814–1.673)	(0.857–1.333)	(1.023–1.773)	(0.498–0.807)	(0.751–1.146)	(0.670–1.203)	(0.463–1.135)
**Disability Status**											
Not Disabled	18,195 (72.19)	Ref.	Ref.	Ref.	Ref.	Ref.	Ref.	Ref.	Ref.	Ref.	Ref.
Disabled	7009 (27.81)	3.242 ***	2.192 ***	2.532 ***	1.665 ***	1.097	1.219	0.637 ***	1.240 **	1.711 ***	1.378 *
		(2.750–3.822)	(1.703–2.822)	(2.149–2.984)	(1.224–2.266)	(0.913–1.319)	(0.958–1.553)	(0.532–0.763)	(1.040–1.479)	(1.353–2.162)	(0.968–1.963)
**Race Group**											
White	20,645 (81.91)	Ref.	Ref.	Ref.	Ref.	Ref.	Ref.	Ref.	Ref.	Ref.	Ref.
Black	705 (2.80)	1.117	0.880	1.411 **	1.286	1.357 *	1.242	1.219	1.699 ***	1.347	1.754 *
		(0.804–1.551)	(0.620–1.251)	(1.023–1.947)	(0.672–2.462)	(0.971–1.897)	(0.792–1.947)	(0.883–1.683)	(1.239–2.329)	(0.823–2.204)	(0.913–3.367)
Latino	1303 (5.17)	1.539 ***	1.049	1.320 **	0.989	1.380 **	0.732	1.837 ***	1.508 ***	1.819 ***	2.073 ***
		(1.196–1.981)	(0.721–1.526)	(1.019–1.710)	(0.643–1.521)	(1.008–1.889)	(0.431–1.241)	(1.386–2.436)	(1.139–1.996)	(1.299–2.547)	(1.366–3.148)
Biracial	1402 (5.56)	1.818 ***	2.734 ***	2.555 ***	1.418 *	1.549 ***	1.482	1.080	1.911 ***	2.111 ***	2.528 ***
		(1.392–2.374)	(2.057–3.635)	(2.004–3.257)	(0.983–2.045)	(1.126–2.132)	(0.921–2.386)	(0.784–1.487)	(1.449–2.519)	(1.404–3.172)	(1.324–4.829)
Other	1149 (4.56)	1.324 *	1.177	1.596 ***	1.079	1.435 *	1.111	0.761 *	1.140	1.230	1.069
		(0.972–1.804)	(0.763–1.816)	(1.190–2.139)	(0.698–1.668)	(0.934–2.204)	(0.588–2.101)	(0.575–1.006)	(0.829–1.569)	(0.772–1.961)	(0.672–1.701)

*n* = 25,204. Ref. indicates reference category (odds ratio of 1). Odds ratios were obtained using complex survey weights. 95% confidence intervals are in parenthesis. *** *p* < 0.01, ** *p* < 0.05, * *p* < 0.1.

**Table 3:** Estimates of Odds Ratios in Favor of Facing Adverse Mental Health and Substance Use Outcomes by History of Sex Work.

	Unadjusted Odds Ratios		Adjusted Odds Ratios	
	No History of Sex Work	History of Sex Work	No History of Sex Work	History of Sex Work
**Mental Health Outcomes**				
Serious Psychological Distress	Ref.	1.771 ***	Ref.	1.437 ***
		(1.455–2.156)		(1.180–1.749)
Ever Had Serious Suicidal Thoughts	Ref.	1.919 ***	Ref.	1.669 ***
		(1.475–2.497)		(1.259–2.212)
Ever Attempted Suicide	Ref.	2.568 ***	Ref.	2.091 ***
		(2.115–3.118)		(1.708–2.560)
Ever Had More than One Suicide Attempt (*n* = 9868)	Ref.	1.587 **	Ref.	1.423 **
		(1.064–2.367)		(1.028–1.971)
**Substance Use Outcomes**				
Current Tobacco Use	Ref.	2.160 ***	Ref.	1.884 ***
		(1.762–2.647)		(1.527–2.325)
Daily Tobacco Use	Ref.	2.201 ***	Ref.	1.945 ***
		(1.713–2.828)		(1.498–2.524)
Binge Drinking	Ref.	1.599 ***	Ref.	1.642 ***
		(1.296–1.972)		(1.351–1.996)
Marijuana Use	Ref.	2.642 ***	Ref.	2.319 ***
		(2.160–3.232)		(1.925–2.793)
Other Illicit Drug Use	Ref.	3.282 ***	Ref.	2.870 ***
		(2.556–4.215)		(2.283–3.608)
Binge Drinking + non-Marijuana Illicit Drug Use	Ref.	3.922 ***	Ref.	3.611 ***
		(2.816–5.463)		(2.706–4.819)

Odds ratios were obtained using complex survey weights. 95% confidence intervals are in parenthesis. *** *p* < 0.01, ** *p* < 0.05. Ref. indicates the reference category (odds ratio of 1). The adjusted odds ratios were obtained by accounting for age group (18–24, 25–44, 45–64, 65+), sexual orientation (heterosexual/straight, not heterosexual/straight), gender identity (man/trans man, woman/trans woman, non-binary/genderqueer, cross-dresser), marital status (married, widowed/divorced/separated, never married), census region (Northeast, Midwest, South, West), education level (high school or less, some college/associate’s degree, bachelor’s degree, graduate or professional degree), income level (no income, $1–9999, $10,000–$24,999, $25,000–$49,999, $50,000+), employment status (employed, unemployed, out of labor force), disability status (disabled, not disabled), and race group (white, black, Latino, biracial, other).

**Table 4:** Share of Respondents with History of Sex Work for Money Reporting Adverse Mental Health and Substance Use Outcomes by Sex Work Industry.

Type of Sex Work	*n* (%)	Serious Psychological Distress	Ever Had Serious Suicidal Thoughts	Ever Attempted Suicide	Ever Had More than One Suicide Attempt	Current Tobacco Use	Daily Tobacco Use	Binge Drinking	Marijuana Use	Other Illicit Drug Use	Binge Drinking + Non-Marijuana Illicit Drug Use
Street-Based Sex Work	410 (15.79)	41.185	80.803	61.687	77.112	39.812	23.721	28.671	38.863	16.053	7.170
		(27.871–54.498)	(71.932–89.675)	(49.824–73.549)	(60.006–94.218)	(27.808–51.817)	(13.848–33.594)	(16.961–40.380)	(26.122–51.603)	(9.247–22.860)	(3.931–10.409)
Sex Work Advertised Online	832 (32.05)	38.375	80.850.	59.962	70.988	41.565	25.413	34.860.	48.056	20.906	14.229
		(28.782–47.969)	(71.986–89.715)	(49.436–70.487)	(52.234–89.743)	(31.317–51.813)	(16.659–34.166)	(25.054–44.666)	(37.061–59.052)	(13.280–28.532)	(7.054–21.404)
Sex Work Advertised in Magazines/Newspapers	110 (4.24)	20.623	79.675	64.156	49.605	31.633	19.259	14.861	36.706	11.289	5.588
		(5.655–35.591)	(64.040–95.310)	(41.793–86.520)	(7.721–91.489)	(10.496–52.770)	(2.442–36.076)	(4.799–24.923)	(13.539–59.873)	(2.885–19.693)	(0.340–10.837)
Informal Sex Work through Word of Mouth	981 (37.79)	52.808	88.362	67.974	89.944	35.715	22.807	37.830.	40.111	19.287	12.449
		(42.550–63.065)	(84.244–92.479)	(60.261–75.686)	(85.584–94.304)	(27.216–44.213)	(15.932–29.682)	(28.410–47.249)	(30.718–49.504)	(12.564–26.011)	(6.429–18.469)
Escort/Call Girl/Rent Boy with an Agency	260 (10.02)	34.222	76.602	56.106	83.729	51.717	33.220.	33.277	44.009	24.609	14.110.
		(23.190–45.255)	(65.268–87.937)	(43.848–68.363)	(71.758–95.700)	(39.439–63.995)	(20.577–45.864)	(22.053–44.501)	(31.530–56.488)	(13.583–35.635)	(4.505–23.714)
Pornography/Picture or Video	802 (30.89)	48.419	86.851	60.494	84.831	39.940.	22.997	30.892	47.771	17.168	9.097
		(39.992–56.845)	(78.730–94.972)	(51.586–69.401)	(79.685–89.978)	(31.385–48.495)	(14.558–31.437)	(24.255–37.529)	(39.358–56.185)	(12.758–21.579)	(6.029–12.166)
Phone Sex	359 (13.83)	49.041	92.032	68.965	91.779	33.783	22.522	29.482	35.440.	15.402	11.198
		(30.200–67.881)	(86.741–97.324)	(54.525–83.406)	(86.193–97.366)	(18.331–49.234)	(8.781–36.264)	(14.764–44.201)	(19.625–51.256)	(4.344–26.461)	(0.511–21.885)
Webcam Work	992 (38.21)	57.875	91.150.	60.654	85.117	41.748	20.169	35.038	46.295	19.003	10.074
		(50.908–64.842)	(87.197–95.103)	(53.350–67.957)	(79.962–90.272)	(34.446–49.051)	(14.943–25.395)	(28.152–41.924)	(39.087–53.504)	(14.068–23.938)	(6.941–13.206)
Erotic Dancer/Stripper	279 (10.75)	23.457	69.518	45.049	75.336	38.746	14.725	25.937	31.591	11.890.	3.895
		(13.932–32.982)	(53.379–85.658)	(30.758–59.340)	(58.737–91.935)	(24.449–53.042)	(4.806–24.645)	(14.295–37.578)	(19.789–43.392)	(3.600–20.180)	(1.602–6.187)
Fetish Work (Dom, Sub, Switch)	690 (26.58)	50.807	87.707	64.826	88.312	41.396	25.062	33.947	49.323	21.919	14.473
		(41.659–59.954)	(81.776–93.638)	(55.416–74.236)	(84.027–92.597)	(32.401–50.391)	(16.515–33.610)	(24.836–43.059)	(40.165–58.482)	(13.163–30.675)	(5.680–23.265)
Other	264 (10.17)	36.368	86.772	59.268	87.300.	51.725	38.177	27.940.	42.509	24.766	9.292
		(24.389–48.348)	(79.528–94.015)	(46.180–72.356)	(79.501–95.100)	(38.174–65.276)	(22.996–53.358)	(17.082–38.798)	(28.072–56.946)	(8.993–40.538)	(3.348–15.236)

Total number of respondents who reported sex work for money = 2596. Estimates were obtained using complex survey weights. 95% confidence intervals shown in parenthesis.

**Table 5:** Adverse Experiences at Drug/Alcohol Facility by History of Sex Work for any Reason. Total number of respondents who visited drug and alcohol treatment facilities = 401. Percentages add to 100% across rows.

	Adverse Experience	No Adverse Experience	Total
No History of Sex Work	24 (11.16%)	191 (88.84%)	215 (100%)
History of Sex Work	49 (26.34%)	137 (73.66%)	186 (100%)
chi^2^ Pr = 0.000			

## Data Availability

The dataset used in this study is available upon request through the Inter-university Consortium for Political and Social Research (ICPSR): https://www.icpsr.umich.edu/web/RCMD/studies/37229/versions/V1 (accessed via data use agreement with the National Center for Transgender Equality approved 3 May 2018).

## References

[1] Meerwijk E.L., Sevelius J.M. (2017). Transgender Population Size in the United States: A Meta-Regression of Population-Based Probability Samples. Am. J. Public Health.

[2] White Hughto J.M., Reisner S.L., Pachankis J.E. (2015). Transgender stigma and health: A critical review of stigma determinants, mechanisms, and interventions. Soc. Sci. Med..

[3] Zhu X., Gao Y., Gillespie A., Xin Y., Qi J., Ou J., Zhong S., Peng K., Tan T., Wang C. (2019). Health care and mental wellbeing in the transgender and gender-diverse Chinese population. Lancet Diabetes Endocrinol..

[4] Crissman H.P., Stroumsa D., Kobernik E.K., Berger M.B. (2019). Gender and Frequent Mental Distress: Comparing Transgender and Non-Transgender Individuals’ Self-Rated Mental Health. J. Womens Health.

[5] Bockting W.O., Miner M.H., Swinburne Romine R.E., Hamilton A., Coleman E. (2013). Stigma, mental health, and resilience in an online sample of the US transgender population. Am. J. Public Health.

[6] Buchting F.O., Emory K.T., Scout, Kim Y., Fagan P., Vera L.E., Emery S. (2017). Transgender Use of Cigarettes, Cigars, and E-Cigarettes in a National Study. Am. J. Prev. Med..

[7] Day J.K., Fish J.N., Perez-Brumer A., Hatzenbuehler M.L., Russell S.T. (2017). Transgender Youth Substance Use Disparities: Results from a Population-Based Sample. J. Adolesc. Health.

[8] Lombardi E. (2007). Substance Use Treatment Experiences of Transgender/Transsexual Men and Women. J. LGBT Health Res..

[9] Sperber J., Landers S., Lawrence S. (2005). Access to Health Care for Transgendered Persons: Results of a Needs Assessment in Boston. Int. J. Transgenderism.

[10] Klein A., Mountz S., Bartle E. (2018). Factors Associated with Discrimination in Social-Service Settings among a Sample of Transgender and Gender-Nonconforming Adults. J. Soc. Soc. Work. Res..

[11] Kattari S.K., Walls N.E., Whitfield D.L., Langenderfer Magruder L. (2017). Racial and Ethnic Differences in Experiences of Discrimination in Accessing Social Services among Transgender/Gender-Nonconforming People. J. Ethn. Cult. Divers. Soc. Work..

[12] Kattari S.K., Walls N.E., Speer S.R. (2017). Differences in Experiences of Discrimination in Accessing Social Services among Transgender/Gender Nonconforming Individuals by (Dis)Ability. J. Soc. Work. Disabil. Rehabil..

[13] Sawicki D.A., Meffert B.N., Read K., Heinz A.J. (2019). Culturally Competent Health Care for Sex Workers: An Examination of Myths That Stigmatize Sex-Work and Hinder Access to Care. Sex Relatsh. Ther..

[14] Benoit C., McCarthy B., Jansson M. (2015). Stigma, sex work, and substance use: A comparative analysis. Sociol. Health Illn..

[15] Deering K.N., Amin A., Shoveller J., Nesbitt A., Garcia-Moreno C., Duff P., Argento E., Shannon K. (2014). A Systematic Review of the Correlates of Violence against Sex Workers. Am. J. Public Health.

[16] Sprankle E., Bloomquist K., Butcher C., Gleason N., Schaefer Z. (2018). The Role of Sex Work Stigma in Victim Blaming and Empathy of Sexual Assault Survivors. Sex. Res. Soc. Policy.

[17] Rouhani S., Decker M.R., Tomko C., Silberzahn B., Allen S.T., Park J.N., Footer K.H.A., Sherman S.G. (2021). Resilience among Cisgender and Transgender Women in Street-Based Sex Work in Baltimore, Maryland. Womens Health Issues.

[18] Park J.N., Decker M.R., Bass J.K., Galai N., Tomko C., Jain K.M., Footer K.H.A., Sherman S.G. (2021). Cumulative Violence and PTSD Symptom Severity Among Urban Street-Based Female Sex Workers. J. Interpers. Violence.

[19] Puri N., Shannon K., Nguyen P., Goldenberg S.M. (2017). Burden and correlates of mental health diagnoses among sex workers in an urban setting. BMC Womens Health.

[20] Ulibarri M.D., Hiller S.P., Lozada R., Rangel M.G., Stockman J.K., Silverman J.G., Ojeda V.D. (2013). Prevalence and Characteristics of Abuse Experiences and Depression Symptoms among Injection Drug-Using Female Sex Workers in Mexico. J. Environ. Public Health.

[21] Young A.M., Boyd C., Hubbell A. (2000). Prostitution, Drug Use, and Coping with Psychological Distress. J. Drug Issues.

[22] Lancaster K.E., MacLean S.A., Lungu T., Mmodzi P., Hosseinipour M.C., Hershow R.B., Powers K.A., Pence B.W., Hoffman I.F., Miller W.C. (2018). Socioecological Factors Related to Hazardous Alcohol use among Female Sex Workers in Lilongwe, Malawi: A Mixed Methods Study. Subst. Use Misuse.

[23] Amogne M.D., Agardh A., Abate E., Ahmed J., Asamoah B.O. (2021). Determinants and consequences of heavy episodic drinking among female sex workers in Ethiopia: A respondent-driven sampling study. PLoS ONE.

[24] Sausa L.A., Keatley J., Operario D. (2007). Perceived risks and benefits of sex work among transgender women of color in San Francisco. Arch. Sex. Behav..

[25] Nadal K.L., Davidoff K.C., Fujii-Doe W. (2014). Transgender Women and the Sex Work Industry: Roots in Systemic, Institutional, and Interpersonal Discrimination. J. Trauma Dissociation.

[26] Roche K., Keith C. (2014). How stigma affects healthcare access for transgender sex workers. Br. J. Nurs..

[27] Scheim A.I., Bauer G.R., Shokoohi M. (2017). Drug use among transgender people in Ontario, Canada: Disparities and associations with social exclusion. Addict. Behav..

[28] Scheim A.I., Bauer G.R., Shokoohi M. (2016). Heavy episodic drinking among transgender persons: Disparities and predictors. Drug Alcohol Depend..

[29] Nuttbrock L., Bockting W., Rosenblum A., Hwahng S., Mason M., Macri M., Becker J. (2014). Gender abuse, depressive symptoms, and substance use among transgender women: A 3-year prospective study. Am. J. Public Health.

[30] Yi S., Chann N., Chhoun P., Tuot S., Mun P., Brody C. (2020). Social marginalization, gender-based violence, and binge drinking among transgender women in Cambodia. Drug Alcohol Depend..

[31] Virupaksha H.G., Muralidhar D., Ramakrishna J. (2016). Suicide and Suicidal Behavior among Transgender Persons. Indian J. Psychol. Med..

[32] Bazargan M., Galvan F. (2012). Perceived discrimination and depression among low-income Latina male-to-female transgender women. BMC Public Health.

[33] Marshall B.D.L., Socías M.E., Kerr T., Zalazar V., Sued O., Arístegui I. (2016). Prevalence and Correlates of Lifetime Suicide Attempts among Transgender Persons in Argentina. J. Homosex..

[34] James S., Herman J., Rankin S., Keisling M., Mottet L., Anafi M. (2016). The Report of the 2015 US Transgender Survey.

[35] Miller W.M., Miller W.C., Barrington C., Weir S.S., Chen S.Y., Emch M.E., Pettifor A.E., Paz-Bailey G. (2020). Sex work, discrimination, drug use and violence: A pattern for HIV risk among transgender sex workers compared to MSM sex workers and other MSM in Guatemala. Glob. Public Health.

[36] Park J.N., Gaydos C.A., White R.H., Decker M.R., Footer K.H.A., Galai N., Silberzahn B.E., Riegger K., Morris M., Huettner S.S. (2019). Incidence and Predictors of Chlamydia, Gonorrhea and Trichomonas Among a Prospective Cohort of Cisgender Female Sex Workers in Baltimore, Maryland. Sex. Transm. Dis..

[37] Argento E., Taylor M., Jollimore J., Taylor C., Jennex J., Krusi A., Shannon K. (2016). The Loss of Boystown and Transition to Online Sex Work: Strategies and Barriers to Increase Safety Among Men Sex Workers and Clients of Men. Am. J. Mens Health.

[38] Ganju D., Saggurti N. (2017). Stigma, violence and HIV vulnerability among transgender persons in sex work in Maharashtra, India. Cult. Health Sex..

[39] Trujillo M.A., Perrin P.B., Sutter M., Tabaac A., Benotsch E.G. (2017). The buffering role of social support on the associations among discrimination, mental health, and suicidality in a transgender sample. Int. J. Transgend..

[40] Treloar C., Stardust Z., Cama E., Kim J. (2021). Rethinking the relationship between sex work, mental health and stigma: A qualitative study of sex workers in Australia. Soc. Sci. Med..

[41] Keuroghlian A.S., Reisner S.L., White J.M., Weiss R.D. (2015). Substance use and treatment of substance use disorders in a community sample of transgender adults. Drug Alcohol Depend..

[42] Hunt J., Bristowe K., Chidyamatare S., Harding R. (2017). ‘They will be afraid to touch you’: LGBTI people and sex workers’ experiences of accessing healthcare in Zimbabwe—an in-depth qualitative study. BMJ Glob. Health.

[43] Scorgie F., Nakato D., Harper E., Richter M., Maseko S., Nare P., Smit J., Chersich M. (2013). ‘We are despised in the hospitals’: Sex workers’ experiences of accessing health care in four African countries. Cult. Health Sex..

[44] Tan R.K.J., Ho V., Sherqueshaa S., Dee W., Lim J.M., Lo J.J.-M., Teo A.K.J., O’Hara C.A., Ong C., Ching A.H. (2021). COVID-19 and the shifting organisation of sex work markets in Singapore. Cult. Health Sex..

[45] Callander D., Meunier É., DeVeau R., Grov C., Donovan B., Minichiello V., Kim J., Duncan D. (2021). Investigating the effects of COVID-19 on global male sex work populations: A longitudinal study of digital data. Sex. Transm. Infect..

[46] Callander D., Thilani Singham Goodwin A., Duncan D.T., Grov C., El-Sadr W., Grant M., Thompson R.J., Simmons M., Oshiro-Brantly J.L., Bhatt K.J. (2022). “What will we do if we get infected?”: An interview-based study of the COVID-19 pandemic and its effects on the health and safety of sex workers in the United States. SSM-Qual. Res. Health.

[47] Platt L., Grenfell P., Meiksin R., Elmes J., Sherman S.G., Sanders T., Mwangi P., Crago A.-L. (2018). Associations between sex work laws and sex workers’ health: A systematic review and meta-analysis of quantitative and qualitative studies. PLOS Med..

[48] Decker M.R., Lyons C., Guan K., Mosenge V., Fouda G., Levitt D., Abelson A., Nunez G.T., Njindam I.M., Kurani S. (2022). A Systematic Review of Gender-Based Violence Prevention and Response Interventions for HIV Key Populations: Female Sex Workers, Men Who Have Sex With Men, and People Who Inject Drugs. Trauma Violence Abus..

[49] Beattie T.S., Bhattacharjee P., Ramesh B.M., Gurnani V., Anthony J., Isac S., Mohan H.L., Ramakrishnan A., Wheeler T., Bradley J. (2010). Violence against female sex workers in Karnataka state, south India: Impact on health, and reductions in violence following an intervention program. BMC Public Health.

[50] (2017). A Systematic Review of Interventions to Reduce Problematic Substance Use Among Transgender Individuals: A Call to Action. Transgender Health.

[51] Nuttbrock L.A. (2012). Culturally Competent Substance Abuse Treatment with Transgender Persons. J. Addict. Dis..

[52] McCullough R., Dispenza F., Parker L.K., Viehl C.J., Chang C.Y., Murphy T.M. (2017). The Counseling Experiences of Transgender and Gender Nonconforming Clients. J. Couns. Dev..

